# Insights on the symbiotic associations of the tea shot hole borer (Coleoptera: Curculionidae)

**DOI:** 10.3389/fmicb.2025.1589710

**Published:** 2025-06-10

**Authors:** Luisa F. Cruz, Octavio Menocal, Christopher Dunlap, Miriam F. Cooperband, Kevin R. Cloonan, Nurhayat Tabanca, Daniel Carrillo

**Affiliations:** ^1^Tropical Research and Education Center, University of Florida, Homestead, FL, United States; ^2^Crop Bioprotection Research Unit, United States Department of Agriculture, Agriculture Research Service, National Center for Agricultural Utilization Research, Peoria, IL, United States; ^3^Forest Pest Methods Laboratory, United States Department of Agriculture, Animal and Plant Health Inspection Service, Plant Protection and Quarantine, Science and Technology, Buzzards Bay, MA, United States; ^4^Subtropical Horticulture Research Station, United States Department of Agriculture, Agriculture Research Service, Miami, FL, United States

**Keywords:** ambrosia beetles, *Euwallacea perbrevis*, fungal farming, *Fusarium*, plant pathogen, Scolytinae, symbiosis

## Abstract

**Introduction:**

The tea shot hole borer (TSHB), *Euwallacea perbrevis* (Schedl 1951) (Coleoptera: Curculionidae) is an invasive ambrosia beetle that carries multiple symbiotic fungi and vectors *Fusarium* spp. to avocado (*Persea americana* Mill.). This study investigated the role of six fungal species (*Fusarium* sp. FL-1, *Fusarium* sp. AF-8, *Fusarium* sp. AF-6, *Graphium* sp., *Acremonium* sp., and *Acremonium murorum*) as nutritional symbionts of TSHB, and the role of *Fusarium* species in plant pathogenicity.

**Methods:**

Four experimental approaches were used: (1) testing each of the six symbionts as a food source for TSHB larvae, (2) examining the stability of symbiotic associations by rearing TSHB on substrates previously colonized by individual fungi, (3) establishing TSHB colonies with single *Fusarium* symbionts (Mono-*Fusarium* Lines, MFL), (4) testing disease development in avocado trees infested with MFL.

**Results:**

*Fusarium* sp. FL-1 and *Fusarium* sp. AF-8 supported the highest percentage of larval development among the tested fungi. These two fungi persisted in the mycangia of beetles reared on a substrate pre-inoculated with other symbionts. In addition, both fungal species caused the largest lesions in avocado branches. TSHB feeding on the other tested symbionts (*Fusarium* sp. AF-6, *Graphium* sp., *Acremonium* sp. or *Acremonium murorum*) resulted in poor larval development and/or overall reduced reproduction compared to feeding upon *Fusarium* sp. FL-1 and AF-8 and the symbiont blend (control).

**Discussion:**

These findings demonstrate the dual role of *Fusarium* sp. FL-1 and AF-8 as nutritional symbionts of TSHB and as key drivers of pathogenicity in avocado.

## 1 Introduction

Ambrosia beetles (Coleoptera: Curculionidae: Scolytinae and Platypodinae) rely on mutualistic fungi as their principal food source (Farrell et al., 2001; Mueller et al., 2005). Conidia of these mutualistic fungi are housed within cuticular pouches called mycentangia or mycangia, which may be located internally or externally in different parts of the beetle’s body (Six, 2003; Vega and Biedermann, 2020). In the case of *Euwallacea* ambrosia beetles, the mycangia are located at the base of their mandibles (O’Donnell et al., 2015). Along with an assemblage of other microorganisms, including bacteria and yeasts, beetles cultivate their mutualistic fungi on the walls of galleries bored into the woody tissue (xylem) of their host trees (Haanstad and Norris, 1985; Biedermann et al., 2013; Ibarra-Juarez et al., 2020). Among their functions, fungi synthesize sterols, digest wood polymeric compounds, concentrate nutrients, and detoxify plant defense compounds (Kok et al., 1970; Biedermann and Vega, 2020). Frequently, ambrosia beetles are engaged in multipartite symbioses with a primary fungus dominating the gallery gardens and providing the greatest nutritional benefits to the foundress and its offspring (Biedermann et al., 2009), while secondary fungi contribute to beetle development to a lesser extent (Six, 2012). In addition, fungal communities undergo a succession process influenced by active farming, resulting in different beetle life stages utilizing distinct symbionts present in the gallery at any given time (Freeman et al., 2016; Cruz et al., 2018).

Although most ambrosia beetles have obligate nutritional relationships with Ophiostomatales (*Raffaelea*) and Microascales (*Ambrosiella*) fungi, the genus *Fusarium* (Hypocreales) also contains species of mutualistic fungi (Dreaden et al., 2014; Mayers et al., 2015; O’Donnell et al., 2016). Ambrosia beetles in the *Euwallacea fornicatus* complex, also known as shot hole borers (SHB), are native to South and Southeast Asia but have invaded several areas in the Americas, Europe, Africa and Australia (Gomez et al., 2018; Smith et al., 2019), and have symbiotic nutritional relationships with *Fusarium* species within the monophyletic group known as the Ambrosia *Fusarium* Clade (AFC) (O’Donnell et al., 2015). Twenty four phylogenetically distinct species distinguished by the prefix AF followed by a number (AF-1 to AF-24) have been identified in the clade, some of which are phytopathogenic and responsible for plant wilting and dieback (O’Donnell et al., 2015; Aoki et al., 2019; Lynn et al., 2020; Lai et al., 2022). Fungi in the genera *Graphium* (Microascales), *Paracremonium,* and *Acremonium* (Hypocreales) have also been associated with members of the SHB complex, although their symbiotic roles are unknown or they are considered auxiliary fungi (Carrillo et al., 2016; Lynch et al., 2016; Na et al., 2018; Carrillo et al., 2020).

*Fusarium* symbioses have drawn particular attention due to their threat to forest, urban, and agricultural settings (Eskalen et al., 2013; Kasson et al., 2013; Na et al., 2018). Although the SHB complex is mainly associated with the degradation of dead or declining trees in its native area, it has also been found attacking healthy trees and causing considerable damage in invaded areas (Beaver, 1989; Owens et al., 2018). Once transferred to the galleries of host trees by female beetles, *Fusarium* spp. moves in a localized manner from the galleries into the xylem elements and adjacent cells, obstructing water, and mineral transport in the plant, resulting in wilting and individual branch dieback (Umeda et al., 2016). Severe cases involving massive beetle attacks combined with multiple infections of the mutualistic fungi may result in the host tree’s death (Eskalen et al., 2013; Hulcr and Stelinski, 2017). *Euwallacea* beetles are polyphagous with a collective host range including more than 412 plant species in 75 families (Gomez et al., 2019). Notably, avocado (*Persea americana* Mill.) has been the main agricultural crop affected by species in the complex during the last decade, as has been recorded in California, Israel, Australia, Florida, and South Africa (Eskalen et al., 2012; Mendel et al., 2012; Carrillo et al., 2016; O’Donnell et al., 2016; Kendra et al., 2017; Paap et al., 2018).

In Florida, *Euwallacea perbrevis* Schedl, or the tea shot hole borer (TSHB), was first detected in 2002 and later in the avocado-producing area of south Florida in 2012 (Carrillo et al., 2012). In 2016, an outbreak of this species was observed in a single avocado orchard, causing damage to approximately 1,500 trees. Subsequently, an area-wide survey revealed that TSHB was distributed across the entire avocado-producing area. However, only 2 out of 33 surveyed orchards sustained significant damage, despite large beetle populations being recorded (Carrillo et al., 2016). The survey confirmed the association of TSHB with *Fusarium* sp. AF-8 and *Fusarium* sp. AF-6, previously identified by Kasson et al. (2013), and revealed new mutualistic fungi, including an unknown species of *Fusarium* (*Fusarium* sp. FL-1)*, Acremonium* sp., *Acremonium murorum,* and *Graphium* sp. (Carrillo et al., 2016). In 2018, an additional survey in natural areas revealed that native trees such as *Lysiloma latisiliquum* (L.) Bentham (Fabaceae) might serve as a reservoir for TSHB, facilitating its invasion into adjacent avocado groves (Owens et al., 2018).

This study aimed to determine the role of *Fusarium* spp. associated with the TSHB as pathogens of avocado. It also evaluated the nutritional roles of six fungal symbionts previously identified in association with TSHB. This was achieved by: (1) testing each of the six symbionts (*Fusarium* sp. FL-1, *Fusarium* sp. AF-8, *Fusarium* sp. AF-6, *Graphium* sp., *Acremonium* sp., and *Acremonium murorum*) individually as a food source for TSHB larvae; (2) examining the stability of symbiotic associations by rearing TSHB on substrates previously colonized by individual fungi, (3) establishing TSHB colonies with single *Fusarium* symbionts (Mono-*Fusarium* Lines, MFL), (4) testing disease development in avocado trees infested with MFL.

## 2 Materials and methods

### 2.1 Beetle collection and rearing conditions

TSHB females excavated from avocado logs (25°31′31″N; 80°29′07″W) were used to establish a laboratory colony. An avocado sawdust medium poured into 50 mL conical centrifuge tubes was used as the rearing system, as described in Menocal et al. (2017). Foundress females were dipped in 70% (v/v) ethanol for 5–7 s and individually placed into rearing tubes. Four holes were punched on the surface of the medium with a sterile probe to facilitate the beetle’s excavation. Rearing tubes were capped with lids fitted with a 1-cm metallic screen opening for airflow and maintained at 25°C and 75% relative humidity, with a 16:8 L:D photoperiod under laboratory conditions.

### 2.2 Fungal isolates and culturing conditions

Six TSHB fungal associates: *Acremonium murorum, Acremonium* sp., *Fusarium* sp. FL-1, *Fusarium* sp. AF-6 (AF-6), *Fusarium* sp. AF-8 (AF-8) and *Graphium* sp. were obtained from stock cultures isolated and identified in a previous study at the Tropical Fruit Entomology Laboratory, University of Florida (Carrillo et al., 2016). Sequences of the strains used and isolated in this study were deposited in GenBank (*A. murorum*: 28S large ribosomal subunit (LSU) MZ262757, 18S small ribosomal subunit (SSU) MZ262755; *Acremonium* sp.: LSU-MZ262757, SSU-MZ262754; *Fusarium* sp. FL-1 translation elongation factor 1-α (EF1-α)-MZ265349, DNA-directed RNA polymerase subunit 1 (RPB1)- MZ265352, the second-largest subunit of RNA polymerase (RPB2)-MZ265355; AF-6: EF1-α-MZ265351, RBP1-MZ265354, RBP2-MZ265357; AF-8: EF1-α-MZ265350, RBP1-MZ265353, RBP2-MZ265356; and *Graphium* sp.: LSU-MZ262759, SSU-MZ262756). Fungal strains were cultured on potato dextrose agar media (BD Difco, NJ, USA) supplemented with 0.2 g/L streptomycin (PDA^+^) (Fisher Scientific, MA, USA) and on beetle’s rearing media, both in 100 × 15 mm Petri dishes (Fisher Scientific, MA, USA). Agar and rearing media plates were incubated at room temperature for 1 week.

### 2.3 TSHB larval feeding assays

Third instar larvae were obtained by dissecting the avocado sawdust media in 20 day-old rearing tubes. Larvae were surface disinfected with a solution of 2% bleach in Phosphate Buffer Saline (PBS), rinsed twice with sterile water, and then transferred to a one-week-old culture of one of the six mutualistic fungi or the non-mutualistic control *Raffaelea arxii* (Ophiostomatales: Ophiostomataceae). While *R. arxii* is not associated with TSHB, it is known to be a symbiont of other ambrosia beetle species, particularly *Xyleborus* spp. (Saucedo-Carabez et al., 2018; Menocal et al., 2018, 2023). Larval survival, pupation, and adult emergence were recorded for 2 weeks. Two independent experiments were carried out, with 18 and 10 larvae per fungal treatment in the first and second experiments, respectively.

### 2.4 Symbiont fidelity assays

**Figure 1:**
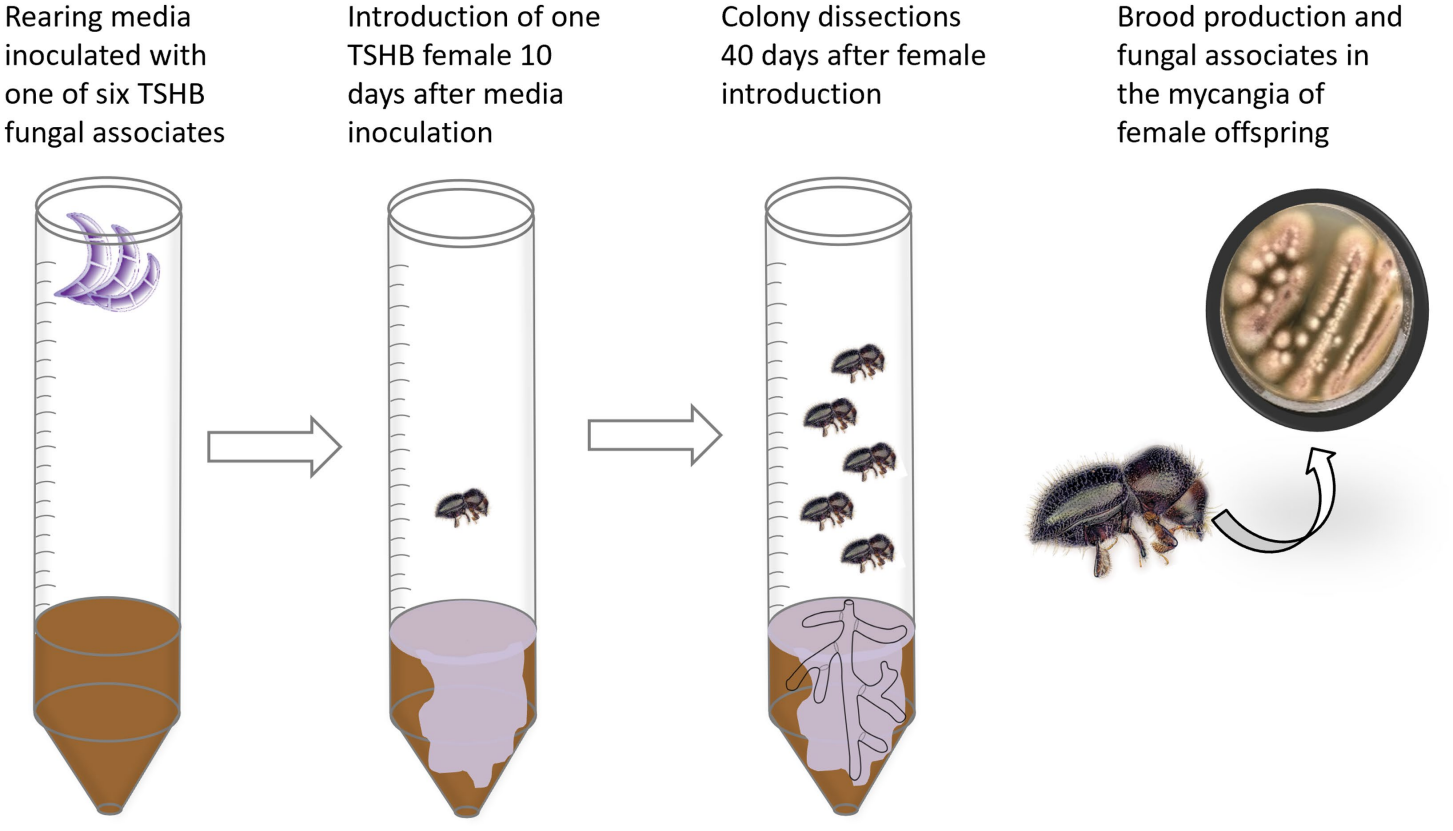
Schematic representation of the methodology used to rear and evaluate TSHB individually inoculated with each of six mutualistic fungi.

To evaluate the stability of symbiotic associations, TSHB females were introduced into rearing tubes with media previously inoculated with 500 μL of a spore solution (1 × 10^6^ CFU) of individual symbionts. Rearing tubes were inoculated per symbiont, and non-inoculated tubes were used as controls. All rearing tubes were incubated at room temperature for 1 week to allow growth of the symbionts before introducing the females. Forty days after the females were introduced, the media were dissected, and all beetle developmental stages were recorded. One mature female per colony was surface disinfected with 70% ethanol for 1 min, rinsed three times with sterile water, and decapitated to identify the fungi in its mycangia (Figure 1). Beetle heads were macerated in 200 μL of sterile water, and subsamples (100 μL) of the head macerates were plated/streaked on PDA^+^ and incubated at room temperature for 1 week. Symbiont abundance and diversity were determined by counting differential morphologies and then molecularly identified as described by Carrillo et al. (2016). The evaluations included the first generation (*n* = 11) reared on pre-inoculated media and the following generation (*n* = 10) on non-inoculated media. Two independent experiments were carried out.

### 2.5 Production of TSHB lines carrying a single *Fusarium* species

TSHB pupae from the stock colony were dipped in 70% ethanol for 30 s, in a 0.01% tween 20 (Sigma-Aldrich, MO, USA) and 2% bleach solution in PBS for 30 s, and then rinsed twice with sterile water. Pupae were then transferred to Petri dishes (100 × 15 mm) containing 7-day-old cultures of one of the three *Fusarium* species grown on the beetle’s rearing media. Ten to fifteen female pupae and two to three male pupae were reared per plate until the adults were completely sclerotized and individually transferred into sterile rearing tubes. TSHB pupae are sexually dimorphic; females are approximately twice the size of males and possess developing wings, while males are wingless. Mycangial fungi from two F_1_ and F_2_ females were isolated and identified as described above to confirm the presence of a single fungal symbiont. The total progeny of each female were recorded in the first and second generations of the MFL.

### 2.6 Tree inoculations with TSHB mono-*Fusarium* lines

**Figure 2:**
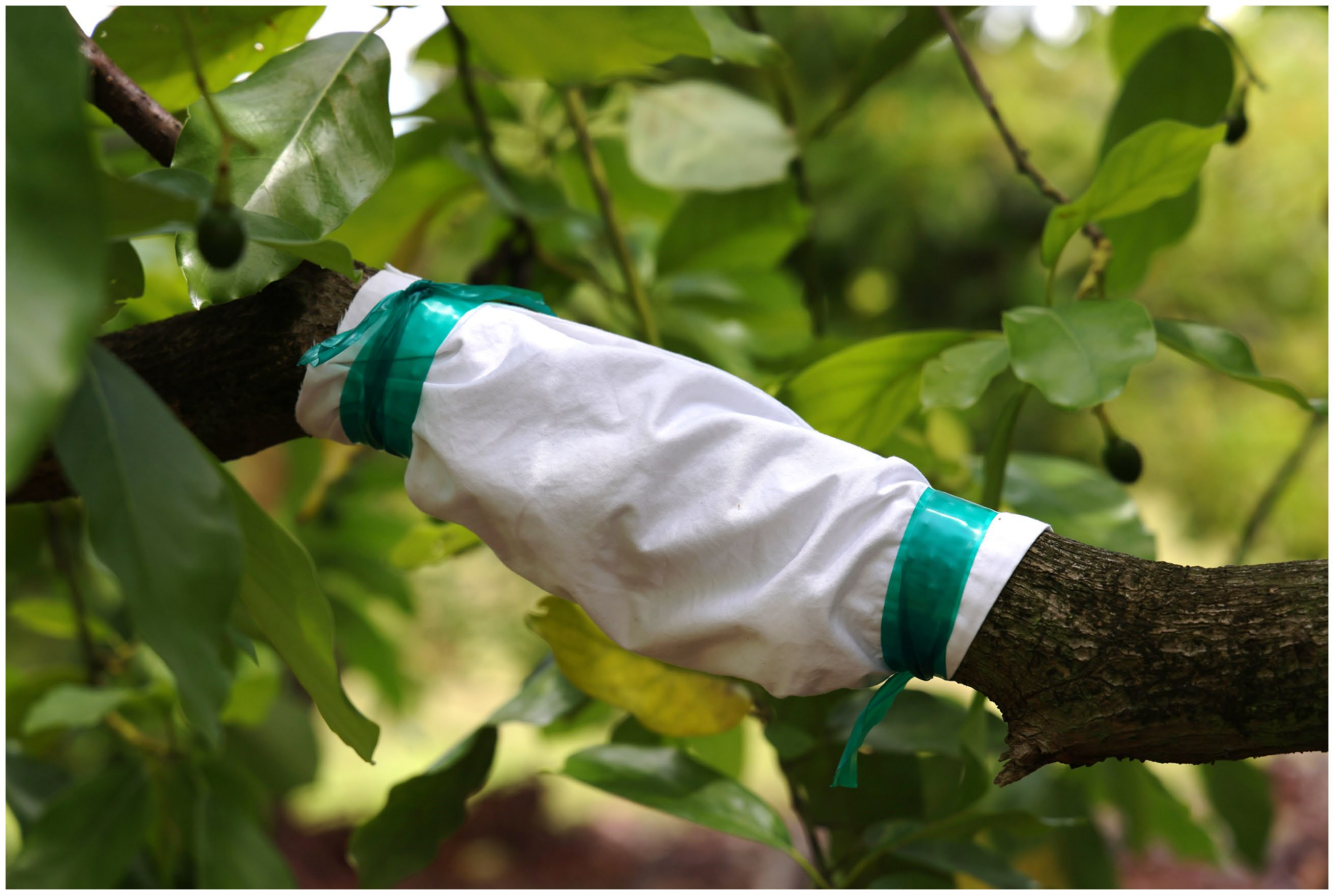
Tree infestation with TSHB mono-*Fusarium* lines. Cohorts of five beetles were used to infest 5-year-old avocado trees (cv. Simmonds’) under field conditions. The beetles were released inside white cotton fabric sleeves affixed to the middle section of the avocado branches with tagging tape.

Cohorts of five beetles from each of the three MFL and a control unmodified laboratory colony were used to infest 5-year-old avocado trees (cv. Simmonds’) under field conditions. The beetles were released inside white cotton fabric sleeves (25 × 15 cm) affixed to the middle section of the avocado branches with 5 mm thick tagging tape (Figure 2). Each TSHB MFL and the control colony were distributed on independent branches of the same tree. A total of 20 trees (replicates) were used in the experiment. Even though no beetle reproduction was observed, trees were inspected weekly for 50 days for disease symptoms. The number and size of lesions caused by fungal infections on the tree branches were determined.

### 2.7 Statistical analyses

Levene’s test under the general linear model procedure (PROC GLM) statement of SAS (Version 9.4, SAS Institute Inc., Cary, NC, USA) was used to assess the equality of variances in the two experimental replications of the symbiont fidelity experiment. Since the variances of the two replications were not statistically different, all data were combined for subsequent analyses. Data from the symbiont fidelity experiment and the MFL were analyzed under the PROC GLM statement. Analysis of variance was performed separately by generation. The experiments followed a completely randomized design, in which fungal treatments were considered a fixed factor. The normal distribution of the residuals was tested under the PROC UNIVARIATE statement. Mean separation analyses were performed using Tukey’s multiple comparison test (*p <* 0.05). The Steel–Dwass method (SAS v. 9.4) was used for non-parametric paired comparisons of mean CFUs of each fungal isolate recovered among the inoculated treatments. Frequencies of fungal recovery and data from the larval feeding assay were analyzed under the PROC GLIMMIX procedure (SAS v. 9.4).

## 3 Results

### 3.1 TSHB larval feeding assays

**Figure 3:**
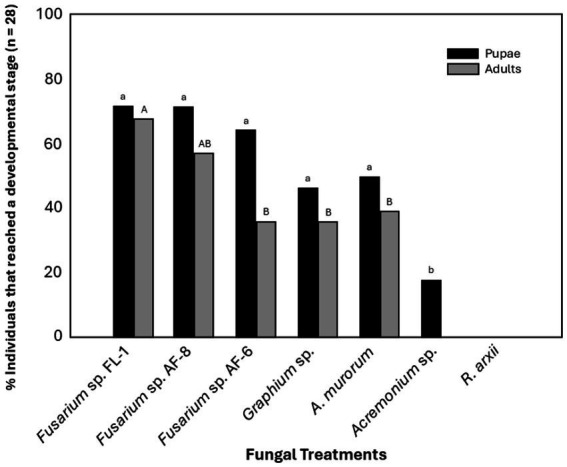
Development of TSHB third instar-larvae reared on different fungal symbionts. Percentage of pupation and individuals reaching adulthood. Bars with the same letter are not significantly different among treatments (*p* < 0.05).

Third instar larvae fed solely on *Fusarium* sp. FL-1 or *Fusarium* sp. AF-8 had the highest pupation rate (71.42%), followed by *Fusarium* sp. AF-6 (64.28%), *A. murorum* (50%), and *Graphium* sp. (46.4%). No significant differences were found among these treatments (Figure 3). The pupation rate of larvae fed on *Acremonium* sp. was significantly lower (17%) (*p* < 0.05). Larvae feeding on *R. arxii* showed 100% mortality by the second day of the experiment (Figure 3). The highest number of adults emerged from larvae fed on *Fusarium* sp. FL-1 (67.85%), followed by intermediate emergence levels on *Fusarium* sp. AF-8 (57.14%), and significantly fewer adults emerged from larvae fed on *Graphium* sp., *A. murorum* (39.3% in both cases) (*p* = 0.0196), and AF-6 (35.71%) (*p* = 0.0365). None of the larvae reared on *Acremonium* sp. reached adulthood (Figure 3).

### 3.2 Symbiont fidelity assays

**Figure 4:**
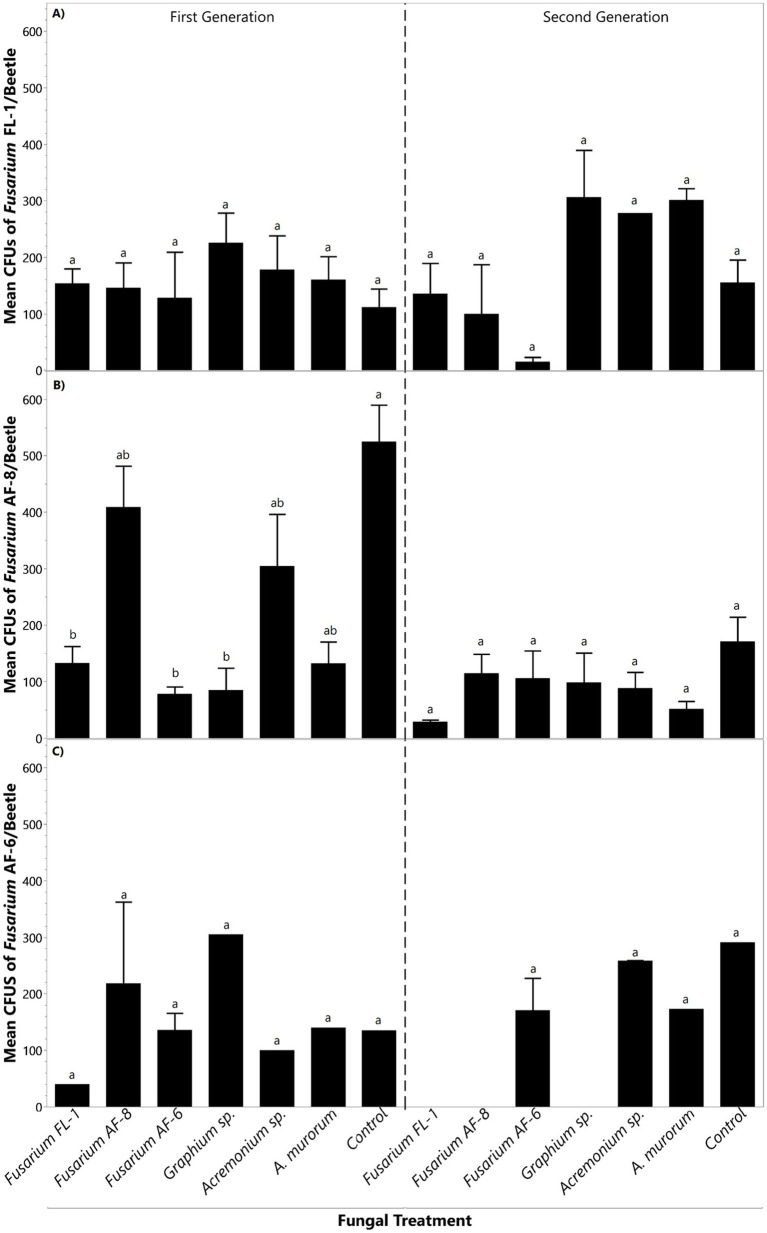
Mean CFU counts of primary symbionts **(A)** *Fusarium* sp. FL-1, **(B)** *Fusarium* sp. AF-8, and **(C)** *Fusarium* sp. AF-6. recovered from beetles’ mycangia within each fungal treatment. The first generation was reared on inoculated media whereas the second generation was reared on non-inoculated media. Bars topped with the same letter within the same generation indicate no significant differences among treatments (*p <* 0.05).

*Fusarium* sp. FL-1 displayed stability across the first and second generations of beetles reared on media inoculated with the different symbionts. Similar numbers of colony-forming units (CFUs) of *Fusarium* sp. FL-1 were recovered from first-and second-generation beetles despite previous inoculation of the rearing media with the other symbionts (Figure 4A).

*Fusarium* sp. AF-8 recovery varied in the first-generation beetles and later stabilized in the second generation. The highest recovery of *Fusarium* sp. AF-8 was from first-generation beetles reared on non-inoculated media (control), followed by those reared on *Fusarium* sp. AF-8, and *Acremonium* sp. (Figure 4B). Beetles reared on *Fusarium* sp. FL-1, *Fusarium* sp., AF-6, *Graphium* sp., and *A. murorum* had significantly fewer CFUs of *Fusarium* sp. AF-8 in the first generation (Figure 4B). No significant differences in *Fusarium* sp. AF-8 CFUs were recorded among fungal treatments in the second generation (Figure 4B).

*Fusarium* sp. AF-6 recovery was similar in first-generation beetles reared on media inoculated with different fungal treatments (Figure 4C). However, *Fusarium* sp. AF-6 was not recovered from second generation beetles previously reared on media inoculated with *Fusarium* sp. FL-1, *Fusarium* sp. AF-8, and *Graphium* sp. (Figure 4C).

**Figure 5:**
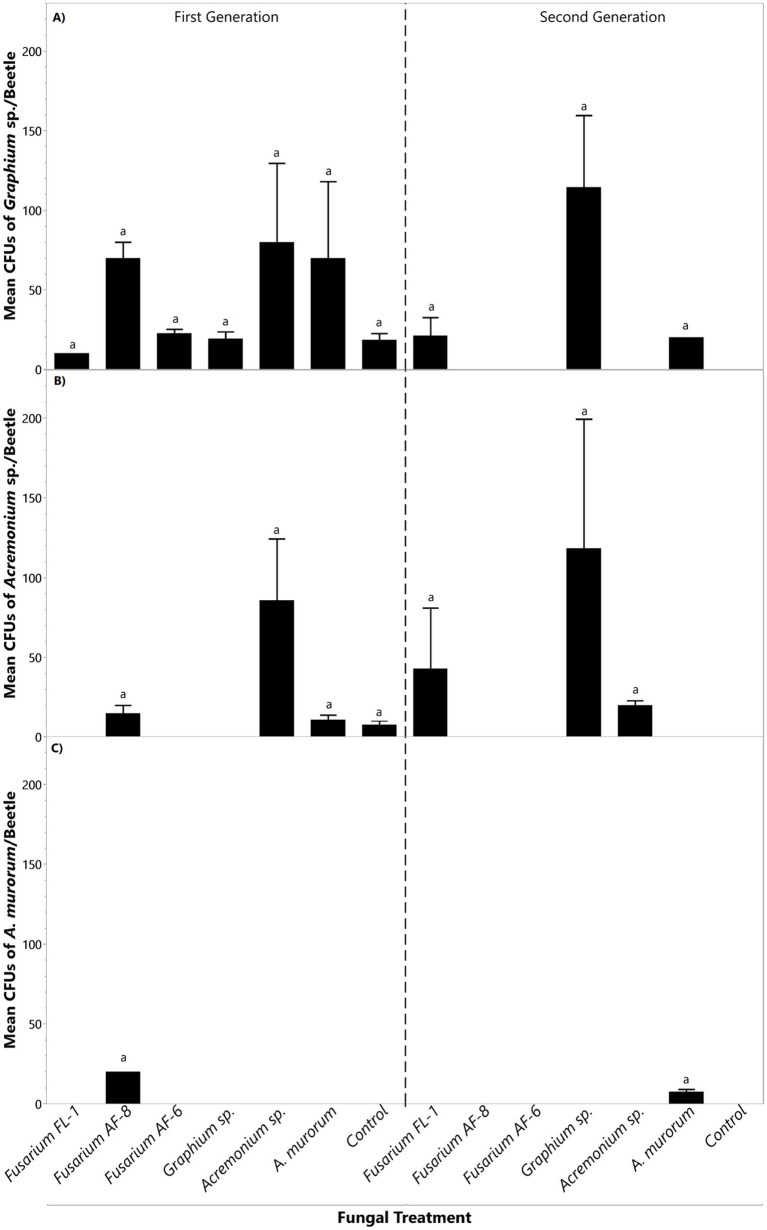
Mean CFU counts of auxiliary symbionts **(A)** *Graphium* sp., **(B)** *Acremonium* sp., and **(C)** *A. murorum* recovered from beetles’ mycangia within each fungal treatment. The first generation was reared on inoculated media whereas the second generation was reared on non-inoculated media. Bars topped with the same letter within the same generation indicate no significant differences among treatments (*p <* 0.05).

*Graphium* sp. recovery was similar in first-generation beetles reared on media with the different fungal treatments (Figure 5A). However, *Graphium* sp. was not recovered from second-generation beetles reared on media previously inoculated with *Fusarium* sp. AF-8, *Fusarium* sp. AF-6, *Acremonium* sp., and the non-inoculated control (Figure 5A).

*Acremonium* sp. was recovered from roughly half of the beetle colonies from the first and second generation beetles exposed to the different fungi. In the first generation, similar CFUs numbers of *Acremonium* sp. were recovered from beetles reared on *Fusarium* sp. AF-8, *Acremonium* sp., *A. murorum*, and the non-inoculated control (Figure 5B). However, *Acremonium* sp. was not detected in beetles reared on *Fusarium* FL-1*, Fusarium* sp., AF-6, and *Graphium* sp. (Figure 5B). Similarly, *Acremonium* sp. was recovered in similar CFU numbers in beetles reared on *Fusarium* sp. FL-1, *Graphium* sp., and *Acremonium* sp. in the second generation (Figure 5B), but it was not recovered from beetles reared on media inoculated with *Fusarium* sp. AF-8, *Fusarium* sp. AF-6, *A. murorum*, and the non-inoculated control (Figure 5B).

*Acremonium murorum* was only recovered from beetles that developed on the *Fusarium* sp. AF-8 and *A. murorum* fungal treatments in the first and second generations, respectively (Figure 5C).

Offspring production in first generation beetles reared on media pre-inoculated with individual symbionts were as follows: *Fusarium* sp. FL-1 (95.4%), *Fusarium* sp. AF-8 (86.4%), *Fusarium* sp. AF-6 (95.4%), *Graphium* sp. (81.8%), *Acremonium* sp. (81.8%), *A. murorum* (86.4%), and control (86.4%). Total brood and female offspring numbers were smaller on all pre-inoculated treatments than on the non-inoculated control (Table 1). The presence of *Fusarium* sp. AF-8 and *Fusarium* sp. AF-6 resulted in fewer females and total brood than with other symbionts (Table 1). Offspring production in second-generation beetles was 50% for *Fusarium* sp. FL-1, 60% for *Fusarium* sp. AF-8, 70% for *Fusarium* sp. AF-6, 75% for *Graphium* sp., 85% for *Acremonium* sp., 55% for *A*. *murorum,* and 80% for the control. The number of females and total brood was significantly smaller in *Fusarium* sp. AF-8 and *Fusarium* sp. AF-6 than in the control and *Acremonium* sp. (Table 1).

**Table 1:** Offspring counts of TSHB reared on media previously inoculated individually with specific fungal symbionts (first generation) and non-inoculated media (second generation).

Fungal treatment	Previously inoculated	N	N offspring	Total brood^§^ (all stages combined)	Adult females	Adult males	Pupae	Larvae	Eggs
First generation									
Control	No	22	19	63.1 ± 3.6 a	35.4 ± 2.8 a	1.9 ± 0.2 a	6.4 ± 0.6 a	12.8 ± 1.7 a	5.2 ± 0.9 a
*Fusarium* sp. FL-1	Yes	22	21	33.1 ± 2.6 b	23.9 ± 2.2 b	1.8 ± 0.2 a	1.6 ± 0.5 cd	3.2 ± 0.9 bc	0.9 ± 0.6 b
*Fusarium* sp. AF-8	Yes	22	19	15.2 ± 2.1 c	11.5 ± 2.0 c	1.3 ± 0.2 ab	0.5 ± 0.2 d	0.7 ± 0.2 c	0.2 ± 0.2 b
*Fusarium* sp. AF-6	Yes	22	21	16.1 ± 2.2 c	11.4 ± 1.6 c	0.9 ± 0.2 b	0.3 ± 0.1 d	1.3 ± 0.5 c	0.5 ± 0.3 b
*Graphium* sp.	Yes	22	18	36.6 ± 3.6 b	21.8 ± 2.4 bc	1.7 ± 0.2 ab	3.9 ± 0.8 b	6.9 ± 1.3 b	0.8 ± 0.7 b
*Acremonium* sp.	Yes	22	18	38.4 ± 4.5 b	26.6 ± 3.1 b	1.7 ± 0.2 ab	2.8 ± 0.8 bc	5.0 ± 1.4 bc	0.3 ± 0.3 b
*A. murorum*	Yes	22	19	26.5 ± 3.6 bc	18.9 ± 3.2 bc	1.3 ± 0.2 ab	0.8 ± 0.3 cd	2.1 ± 0.8 6c	1.1 ± 0.5 b
Second generation									
Control	No	20	16	42.7 ± 6.2 a	39.9 ± 3.3 a	6.6 ± 2.5 a	4.1 ± 0.8 a	2.0 ± 0.7 b	0.3 ± 0.14 a
*Fusarium* sp. FL-1	No	20	10	30.3 ± 3.1 ab	24.4 ± 2.4 abc	1.4 ± 0.2 ab	0	0	0
*Fusarium* sp. AF-8	No	20	12	23.7 ± 3.8 ab	19.0 ± 3.20 cb	0.8 ± 0.2 b	0	0	0.1 ± 0.1 a
*Fusarium* sp. AF-6	No	20	14	18.6 ± 2.7 b	14.4 ± 1.7 c	1.4 ± 0.4 ab	0.1 ± 0.1 c	0	0
*Graphium* sp.	No	20	15	47.2 ± 6.7 a	33.2 ± 4.5 ab	1.6 ± 0.2 ab	3.9 ± 0.8 a	5.5 ± 1.1 a	0
*Acremonium* sp.	No	20	17	42.2 ± 5.5 a	35.1 ± 4.3 a	2.0 ± 0.3 ab	0.9 ± 0.4 bc	1.0 ± 0.3 b	0
*A. murorum*	No	20	11	33.6 ± 6.9 ab	28.6 ± 4.5 abc	4.9 ± 2.2 ab	3.0 ± 1.1 ab	1.5 ± 0.6 b	0

§ Colonies were dissected 40 days after the introduction of the foundresses. Mean ± SE values followed by the same letter within columns are not significantly different among treatments (Tukey’s multiple comparison test; *p* < 0.05).

### 3.3 Production of TSHB lines carrying a single *Fusarium* species

Three TSHB mono-*Fusarium* lines or MFL (i.e., *Fusarium* sp. FL-1, *Fusarium* sp. AF-8, and *Fusarium* sp. AF-6) were successfully established following the methods described above. Forty days after the initiation of the colonies, total brood showed no significant differences among the three MFLs compared to the control. However, F_1_ female numbers were significantly smaller for *Fusarium* sp. AF-6 than the other treatments (*p* = 0.0212). There were significantly fewer larvae in the *Fusarium* sp. FL-1 colony than in other colonies (*p* = 0.0095). The number of pupae and adult males showed no significant differences across the treatments, and no eggs were observed in any of the colonies at this evaluation point (Table 2). In the second generation, the total brood and the number of females were significantly smaller in *Fusarium* sp. AF-6 than the other MFLs and the laboratory colony used as the control (*p* < 0.0001). *Fusarium* sp. AF-6 showed significantly fewer males than the control (*p* = 0.0063); however, no significant differences were found in the number of males between this treatment and the other MFL. The number of pupae was larger in *Fusarium* sp. AF-8 compared to the rest of the MFLs (*p* = 0.0002). *Fusarium* sp. AF-8 and *Fusarium* sp. FL-1. showed significantly larger numbers of larvae than *Fusarium* sp. AF-6 and the control (*p* < 0.0001). The number of eggs showed no differences among treatments (Table 2).

**Table 2:** Number of individuals per developmental stage during two generations of TSHB mono-*Fusarium* lines.

Line	Total brood^§^ (all stages combined)	Adult females	Adult males	Pupae	Larvae	Eggs
First generation						
Control	48.0 ± 5.0 a	36.4 ± 3.8 a	1.60 ± 0.2 a	3.8 ± 1.1 a	6.2 ± 0.7 a	0
*Fusarium* sp. FL-1	43.0 ± 3.0 a	35.2 ± 2.6 a	1.64 ± 0.2 a	3.8 ± 0.9 a	2.4 ± 0.4 b	0
*Fusarium* sp. AF-8	45.4 ± 7.4 a	31.2 ± 4.9 a	1.40 ± 0.2 a	6.2 ± 1.7 a	6.6 ± 1.6 a	0
*Fusarium* sp. AF-6	33.4 ± 2.0 ab	22.6 ± 0.9 b	1.80 ± 0.4 a	5.0 ± 0.9 a	4.0 ± 1.4 ab	0
Second generation						
Control	56.2 ± 5.4 a	41.1 ± 3.9 a	2.2 ± 0.2 a	5.9 ± 1.2 b	7.0 ± 1.3 b	0
*Fusarium* sp. FL-1	67.3 ± 3.1 a	46.3 ± 2.5a	1.7 ± 0.2 ab	6.2 ± 0.9 b	12.0 ± 1.7 a	1.2 ± 0.6 a
*Fusarium* sp. AF-8	65.4 ± 3.7 a	38.6 ± 3.0 a	2.1 ± 0.2 ab	12.0 ± 0.8 a	12.7 ± 1.1 a	0
*Fusarium* sp. AF-6	24.9 ± 2.9 b	13.6 ± 2.2 b	1.3 ± 0.3 b	4.8 ± 1.1 b	3.9 ± 0.7 b	1.3 ± 1.3 a

§ Colonies were dissected 40 days after the introduction of the foundresses. Mean ± SE values followed by the same letter within columns are not significantly different among treatments (Tukey’s multiple comparison test; *p* < 0.05).

### 3.4 Fungal transmission by MFL

Boring attempts or small tunnels were found throughout the branches infested with the three MFLs and the control treatment. However, TSHB reproduction was not observed in any of the MFLs or the control. The greatest numbers of boring attempts and most pronounced xylem discoloration due to the fungal infection were found for *Fusarium* sp. FL-1. and *Fusarium* sp. AF-8. Lesion length (xylem discoloration measured up and down the boring point) was more extensive in the control and *Fusarium* sp. FL-1. than in *Fusarium* sp. AF-8 and *Fusarium* sp. AF-6 (Table 3). None of the infested branches developed wilting symptoms.

**Table 3:** Trees infestation with the mono-*Fusarium* lines.

Mono-*Fusarium* line	Total no. of boring attempts	Total no. of lesions	Average lesion length (cm)	Lesion range (cm)
Control	6	3	9.8	7.5–11.0
*Fusarium* sp. FL-1	15	8	8.0	2.0–17.0
*Fusarium* sp. AF-8	14	10	5.4	1.0–11.0
*Fusarium* sp. AF-6	5	3	4.5	3.0–6.0

## 4 Discussion

The *E. fornicatus* complex mutualistic associations have been reported with very particular lineages of fungi (Carrillo et al., 2016; Freeman et al., 2016; Lynch et al., 2016; Na et al., 2018). While certain levels of plasticity in these associations have been observed in their native range in Southeastern Asia (Carrillo et al., 2019), when introduced in exotic environments, the species in the complex have established nutritional relationships with specific pools of symbionts. The Kuroshio shot hole borer (KSHB) in California is associated with *Fusarium kuroshio* and *Graphium kuroshio* (Na et al., 2018), while PSHB in California and Israel are associated with *Fusarium euwallacea, Graphium euwallacea,* and *Pancremonium pembeum* (Freeman et al., 2016; Lynch et al., 2016). Unlike other invaded areas in Florida, TSHB was found linked with more than one species of *Fusarium:* i.e., AF-8, AF-6, and FL-1, in addition to *Graphium* sp., *Acremonium* sp., and *A. murorum* (Carrillo et al., 2016). In this study, we examined the roles of these fungi in the development and reproduction of TSHB and the contribution of different *Fusarium* spp. in disease development.

*Fusarium* species in the AFC are considered primary symbionts of the fungal farming *Euwallacea* spp. due to their consistent association with the beetle’s mycangia and galleries regardless of the host plant (O’Donnell et al., 2015; Lynch et al., 2016). The dependency of PSHB on *F. euwallacea* as a food source for populations in Israel and California has been demonstrated (Freeman et al., 2012). TSHB larvae reached adulthood feeding solely on any of its *Fusarium* associates. *Fusarium* sp. FL-1 and *Fusarium* sp. AF-8 contributed the most as a nutritional source for immature and adult stages. *Fusarium* sp. AF-6 supported the development of immature stages but resulted in significantly fewer adult females (Table 2). Similarly, the number of F_1_ females recorded for the MFL was significantly smaller for *Fusarium* sp. AF-6 than the other two *Fusarium* species.

Studies on the dynamics of mutualistic symbionts during the life cycle of PSHB indicated late instar larvae and non-sclerotized (teneral) adults preferred *G. euwallacea* as a food source, while first instar larvae and mature females feed primarily on *F. euwallacea* (Freeman et al., 2016). *Graphium* sp. and *A. murorum* are less effective as food sources for TSHB immature stages, and *Acremonium* sp. did not support the development of immature stages at all (Figure 5). Likewise, PSHB and KSHB females fed on *Graphium* spp. or *P. pembeum* produced fewer offspring than those fed on their *Fusarium* symbionts (Carrillo et al., 2020).

*Acremonium* spp. has been reported associated with several bark and ambrosia beetles (Yang et al., 2010; Belhoucine et al., 2011; Tarno et al., 2016; Malacrinò et al., 2017; Tuncer et al., 2018; Leonardi et al., 2024). Species in the genus *Acremonium* are known to produce numerous secondary metabolites with diverse biological functions, including antimicrobial activity and production of volatiles attractive to ambrosia beetles (Tian et al., 2017; Gugliuzzo et al., 2023). *Acremonium* spp. may have a nutritional function, but they may also play a primary role in antagonizing contaminant fungi within the beetle galleries.

To understand symbiont dynamics under natural infections, we determined the effect of substrate pre-inoculation with individual symbionts on the fungal community and beetle reproduction. This occurs when avocado branches suffer from multiple subsequent TSHB infestations. Our results show that despite previous infestation and fungal colonization of the branch, beetles appear to control the number of fungal propagules in their mycangia. The specific mechanisms that drive selectivity in the mycangium are still unknown. Mycangial affinity can be influenced indirectly by the beetles’ selection of nutritional sources that are more suitable for specific fungi. Evidence of active selection through volatiles that act as olfactory cues for distinguishing nutritional fungal symbionts has been demonstrated in this beetle (Kendra et al., 2022). However, fungi may compete within the gallery microenvironment, with faster-growing species or those producing specific secondary metabolites potentially dominating and shaping the composition of the fungal gardens. Our experiments were conducted using a limited number of fungal species, which were artificially cultured and presented to TSHB under controlled conditions. In natural environments, however, a more complex, multipartite symbiotic system exists, modulated by unknown fungi, bacteria, other microorganisms, and the host plant itself. While our experimental approach enabled us to elucidate the roles of selected fungi as nutritional symbionts, it does not capture the full complexity of these interactions, which warrants further investigation.

Alterations of the fungal gardens’ composition may explain the significant decrease in brood production in the first generation (inoculated). The beetle’s reproduction was generally restored in the next-non-inoculated generation, except for the *Fusarium* sp. AF-6 inoculated treatment (Figure 4C). This is expected considering its impact on one of the main nutritional fungi of the beetles, *Fusarium* sp. AF-8. There was also a reduction in the percentage of colony establishment in the second generation, especially in the *Fusarium* spp. treatments. This could result from the change in frequencies observed in the first generation and the absence of *Graphium* sp. and *Acremonium* sp. recorded for these treatments in the second generation. These auxiliary fungi appear suboptimal for offspring production of TSHB but are known to support the life cycle of both PSHB and KSHB (Carrillo et al., 2020).

In this study and Carrillo et al. (2016), *Fusarium* sp. AF-6 was the least frequently recovered *Fusarium* spp. from TSHBs naturally breeding in avocado trees or reared on avocado-sawdust-based media. The inferior performance of *Fusarium* sp. AF-6 as a food source may be explained by its weak/slow growth on avocado wood, as was evident by a smaller lesion size observed from the infestations with the *Fusarium* sp. AF-6 MFL (Table 3). The host or the environmental conditions may serve as a filter for species in the same guild (Wellnitz and Poff, 2001), particularly nutritional symbionts. Therefore, symbionts may perform differently based on their adaptation to the host xylem-sapwood: moisture, nutritional content, secondary metabolites, or defensive compounds. For instance, *Fusarium* sp. AF-9 has been isolated from TSHB galleries in *Lysiloma latisiliquum* (wild tamarind) and *Delonix regia* (royal poinciana) (O’Donnell et al., 2016; Owens et al., 2018), but so far, it has not been recovered from avocado-reared beetles nor their galleries in avocado. Previous studies on fungal communities of PSHB established differences in the relative frequencies of their symbionts when recovered from different hosts (Lynch et al., 2016). These observations suggest various degrees of adaptation of the fungal species to a given host tree species. This trend has also been observed for other ambrosia beetles’ mutualistic fungi (Kendra et al., 2013; Campbell et al., 2016). We suggest the environment, particularly the host substrate, plays a significant role in symbiont growth and, in the resulting mutualistic interaction.

In the experiments involving infestation of avocado branches with individuals from the MFL colonies, inoculation of the corresponding single *Fusarium* sp. was confirmed. However, no beetle reproduction was observed, and regardless of pathogen presence, no wilting symptoms were recorded. This suggests that active fungal cultivation by the beetles within their galleries is necessary for natural fungal growth and disease development. In our experiments, only a small number of beetle boring attempts and minimal inoculation of the fungal pathogens were recorded. Previous studies have reported that multiple attacks of PSHB are required to weaken stems and enable subsequent colony establishment (Mendel et al., 2017). Freeman et al. (2019) also determined that symptom development requires both pathogen inoculation and beetle reproduction activity. The absence of symptoms in our study may also be attributed to the initial physiological condition of the plants, which were healthy and non-stressed.

The roles of fungal mutualistic species are influenced by environmental conditions and the host plant. The functional redundancy of mutualists enables beetles to survive under heterogeneous conditions and minimize the competition between developmental stages for food resources (Six, 2012). In the case of the TSHB, the presence of multiple *Fusarium* species, which are the primary symbionts of this beetle species, may facilitate the colonization of different hosts. For instance, *Fusarium* sp. AF-8 and *Fusarium* sp. FL-1 play a primary role in the nutrition of beetles reared in avocado. The auxiliary fungi may also support beetle development, as seen with *Graphium* sp., or perform additional functions, such as suppressing the growth of unwanted fungi or bacteria, which could be the case with *Acremonium* spp. Finally, our experimental results suggest that the establishment of TSHB colonies in host trees may require multiple beetle attacks to achieve sufficient pathogen inoculation. Further studies are needed to clarify the role of the different *Fusarium* species in pathogenicity and symptom development.

## Data Availability

The raw data supporting the conclusions of this article will be made available by the authors without undue reservation.
